# Anti-C1q Autoantibodies, Novel Tests, and Clinical Consequences

**DOI:** 10.3389/fimmu.2013.00117

**Published:** 2013-05-14

**Authors:** Michael Mahler, Rosanne A. van Schaarenburg, Leendert A. Trouw

**Affiliations:** ^1^INOVA Diagnostics, Inc.San Diego, CA, USA; ^2^Department of Rheumatology, Leiden University Medical CenterLeiden, Netherlands

**Keywords:** C1q, complement, autoantibody, diagnosis, SLE

## Abstract

Although anti-C1q autoantibodies have been described more than four decades ago a constant stream of papers describing clinical associations or functional consequences highlights that anti-C1q antibodies are still hot and happening. By far the largest set of studies focus on anti-C1q antibodies is systemic lupus erythematosus (SLE). In SLE anti-C1q antibodies associate with involvement of lupus nephritis in such a way that in the absence of anti-C1q antibodies it is unlikely that a flare in nephritis will occur. Anti-C1q antibodies occur in several autoimmune conditions but also in healthy individuals. Although considerable progress has been made in the understanding of how anti-C1q antibodies may contribute to tissue injury there is still a lot to learn about the processes involved in the breaking of tolerance to this protein. There has been considerable improvement in the assays employed to test for the presence of anti-C1q antibodies. Hopefully with these new and standardized assays at hand larger clinical association studies will be conducted with independent replication. Such large-scale studies will reveal the true value of clinical testing for anti-C1q autoantibodies in several clinical conditions.

## Introduction

Of all the autoantibodies that target complement proteins, anti-C1q autoantibodies have received most attention (Trouw et al., 2001; Norsworthy and Davies, 2003). C1q, the initiation molecule of the classical pathway of complement activation, has a unique capacity to bind to the Fc tail of subclasses of IgG and IgM antibodies (Daha et al., 2011). C1q does so only when at least two IgG molecules are spatially oriented in such a way that they can simultaneously interact with one C1q molecule as for example in an immune-complex (Cooper, 1985). Alternatively, C1q can bind to a single IgM molecule in a “staple” like configuration (Feinstein et al., 1971). The binding of C1q to non-aggregated IgG molecules or fluid-phase IgM is weak. The fact that C1q can bind IgG in immune complexes can be considered as both a blessing and a curse. The blessing lies in the fact that the identification of anti-C1q antibodies was a consequence of studies on size fractionations of immune complexes that could bind to C1q. In these studies it was subsequently discovered that in systemic lupus erythematosus (SLE) patients next to the high molecular weight fractions also low-molecular weight fractions contained immunoglobulins that could bind to C1q (Agnello et al., 1971) (Table 1). In the following years these low-molecular weight fractions were further identified as monomeric, non-complexed IgG molecules that specifically interacted with the collagen-like tail of the C1q molecule (Uwatoko et al., 1984, 1987; Antes et al., 1988). The curse lies in the fact that special care has to be taken to discriminate between IgG in immune complexes binding to C1q and anti-C1q autoantibodies binding to C1q (Kohro-Kawata et al., 2002). This problem can be overcome by adding 1 M NaCl to the incubation buffer in the assay. The low-avidity interactions between the C1q globular head domains with the CH2 domains of IgG Fc tails is completely disrupted in these conditions, whereas the high avidity binding of anti-C1q autoantibodies to the collagen-like tail of C1q is kept intact (Kohro-Kawata et al., 2002). It is currently not known to what extend the high salt conditions may lead to an underestimation of low-avidity anti-C1q autoantibodies.

**Table 1:** **History of anti-C1q antibodies**.

Year	Milestone	Reference
1971/88	Identification of C1q as the target of autoantibodies	Agnello et al. (1971), Antes et al. (1988)
1982	C1q in solid-phase exposes neo-epitopes	Golan et al. (1982)
1984	Identification of the collagen-like stalk as the main binding site of anti-C1q antibodies	Uwatoko et al. (1984, 1987)
1987	Anti-C1q associates with the occurrence of LN	Wener et al. (1987); reviewed a. o. Trendelenburg (2005)
1991	C1q and anti-C1q are enriched in the glomeruli of LN	Uwatoko et al. (1991)
1996	Identification of anti-C1q in mice	Hogarth et al. (1996)
1993	Anti-C1q also present in healthy population and increase with age	Siegert et al. (1993)
2004	Experimental evidence on how anti-C1q can be pathogenic to the kidney in LN but not in healthy individuals	Trouw et al. (2004a)
2007	Identification of anti-C1q antibodies that target the globular heads	Tsacheva et al. (2007)

## Assays to Detect Anti-C1q Autoantibodies

Over time several assays have been developed to detect anti-C1q autoantibodies both in humans and in experimental animal models. The first assays employed a direct coating of intact C1q, which necessitated the use of high salt conditions to discriminate between immune-complex binding and anti-C1q autoantibody binding (Kohro-Kawata et al., 2002). Already early in the history of anti-C1q autoantibodies it was discovered that the majority of these autoantibodies is directed against the collagen-like part of the C1q molecule (Antes et al., 1988). From equilibrium studies and from the observation that anti-C1q antibodies can be found in the presence of freely circulating C1q it was argued that anti-C1q antibodies may interact with epitopes that are not exposed in C1q in fluid phase (Golan et al., 1982). Later these arguments were supported by elegant studies using phage display technology generated Fab fragments that only interacted with solid-phase C1q (Schaller et al., 2009). Next, assays have been developed that utilized only the C1q collagen-like region, generated by enzymatic digestions as antigen (Antes et al., 1988; Wener et al., 1989). This eliminated the need to use high-ionic strength buffer. A recent paper reports on the use of peptides derived from C1q that have interesting properties to detect a major linear epitope in a high percentage of the patients in the absence of high-ionic strength buffer (Vanhecke et al., 2012). In contrast to the assays reported before that anti-C1q antibodies only target the collagen-like region of C1q in 2007 it was discovered that there are also antibodies that specifically target the globular head regions of C1q (Tsacheva et al., 2007).

To study anti-C1q antibodies in experimental animal models, assays were developed that used coating of purified mouse C1q and high salt conditions similar to the human situation (Hogarth et al., 1996; Trouw et al., 2003). Next, in order to circumvent the purification of mouse C1q, an assay was developed which employed a coating of C1q binding peptides, that captured C1q from Rag^−/−^ serum, as the antigenic entity for the anti-C1q ELISA (Trouw et al., 2004a,b).

Several commercial assays are available for the detection of anti-C1q antibodies (see Table 2). Those assays are mostly based on the ELISA technology and are marketed by Bühlmann Laboratories AG (CH-4124 Schönenbuch, Switzerland), IMTEC (HUMAN, Wiesbaden, Germany), Orgentec (Mainz, Germany), and INOVA Diagnostics (San Diego, CA, USA). The ALEGRIA system is a semi-automated assay system based on patient specific modified microtiter plate strips. Some of the anti-C1q antibody assays have been used in clinical studies (Trendelenburg et al., 2006; Potlukova and Kralikova, 2008; Heidenreich et al., 2009; Meyer et al., 2009; Cai et al., 2010; Akhter et al., 2011; Julkunen et al., 2012). Until today, none of the anti-C1q antibody assays achieved clearance by the Food and Drug Administration (FDA) due to the lack of prospective studies. In addition, systematic studies comparing anti-C1q antibody assays from different companies are missing.

**Table 2:** **Overview of commercially available assays for the detection of anti-C1q antibodies**.

Trade name (P/N)	Company	Technology	Reference
Anti-C1q autoantibody ELISA (EK-AC1QA)	Buehlmann Laboratories AG	ELISA	Trendelenburg et al. (2006), Potlukova et al. (2008), Meyer et al. (2009), Julkunen et al. (2012)
IMTEC-anti-C1q-antibodies (ITC59033)	IMTEC	ELISA	Cai et al. (2010)
QUANTA lite anti-C1q (704565)	INOVA Diagnostics	ELISA	Akhter et al. (2011)
Anti-C1q (ORG 549)	Orgentec	ELISA	Heidenreich et al. (2009), Julkunen et al. (2012)
Anti-C1q (ORG 249)	Orgentec	Alegria	NA

## Occurrence of Anti-C1q Autoantibodies and Clinical Associations

Over the past four decades anti-C1q autoantibodies have been studied in a wide variety of autoimmune and renal conditions as well as in infectious diseases (Trendelenburg, 2005). In the healthy population the prevalence of anti-C1q autoantibodies ranges between 2 and 8% (Wener et al., 1989; Siegert et al., 1992a; Trendelenburg et al., 1999; Horvath et al., 2001a; Potlukova et al., 2008) and increases with age (Siegert et al., 1993). Hypocomplementemic Urticarial Vasculitis Syndrome (HUVS) represents the clinical condition with the highest percentage of anti-C1q positivity; 100% (Wisnieski and Jones, 1992). Other conditions characterized by high anti-C1q antibody prevalence are, mixed connective tissue disease (94%), Felty’s syndrome (76%), and SLE (30–60%) (Siegert et al., 1992a; Trendelenburg, 2005; Potlukova et al., 2008; Sinico et al., 2009). The occurrence of anti-C1q autoantibodies was shown to have familial clustering, indicating that there is a genetic risk factor that together with environmental cues may precipitate the production of these antibodies (Hunnangkul et al., 2008). Anti-C1q autoantibodies have also been described to occur in infectious diseases although at a frequency of around, for example 13% of HIV infected individuals vs. 5% in healthy controls (Prohaszka et al., 1999) or up to 26% in patients suffering from hepatitis C virus infection as compared to 10% of healthy controls (Saadoun et al., 2006).

Especially the association between anti-C1q antibodies and renal involvement in SLE has received much attention (Seelen et al., 2003). Correct diagnosis of a flare of lupus nephritis (LN) still represents an important challenge. Serological identification of a flare would be preferred over repeated renal biopsy. Although several anti-nuclear antibodies are associated with renal involvement and active disease (Heidenreich et al., 2009), the presence of anti-C1q antibodies either alone or in combination with other serological markers is superior to predict/correlate with active LN as reviewed before (Trendelenburg, 2005; Sinico et al., 2009). Several studies provide evidence that anti-C1q autoantibodies are superior to other serological markers in identifying a flare of LN (Mok et al., 2010; Akhter et al., 2011). However, other studies indicate that combinations of anti-C1q antibodies with other serological markers are superior to anti-C1q antibodies alone (Matrat et al., 2011; Julkunen et al., 2012; Yang et al., 2012). Especially striking is the strong negative predictive value of anti-C1q testing for LN. In the absence of anti-C1q autoantibodies it is very unlikely that a patient with LN will develop a flare (Trendelenburg et al., 1999, 2006; Meyer et al., 2009; Mok et al., 2010; Matrat et al., 2011; Moura et al., 2011). As many of these studies report on rather small patient populations from very diverse ethnic backgrounds ranging from Brazil (Moura et al., 2011), China (Zhang et al., 2011), India (Pradhan et al., 2012), and Egypt (ElGendi and El-Sherif, 2009) it is likely that considerable variation exists in the strength at which anti-C1q antibodies are associated with and is predictive for LN flares. Several of the larger studies from Europe and Hong Kong point in the same direction (Moroni et al., 2001; Mok et al., 2010; Julkunen et al., 2012) and also a recent meta-analysis confirmed the diagnostic value of serum anti-C1q antibodies for LN (Yin et al., 2012).

Whether or not anti-C1q antibodies are also associated with the disease activity of LN remains to be established as currently there is no consensus on this issue (Horvath et al., 2001b; Grootscholten et al., 2007; Julkunen et al., 2012). One mechanism to clear anti-C1q autoantibodies from the circulation is to use immunoabsorption on C1q-columns (Hiepe et al., 1999). This method depleted next to circulating immune complexes also anti-C1q autoantibodies and was shown to be beneficial in SLE patients (Berner et al., 2001; Pfueller et al., 2001).

## Anti-C1q Antibodies as Part of Multiplex Technologies

With the emerging availability of multiplex technologies for the detection of autoantibodies and other serological markers, the combination of anti-C1q antibodies with other markers is facilitated (Papp et al., 2012). Recent studies have demonstrated that biomarker profiles have the potential to improve the diagnosis of SLE (Kalunian et al., 2012).

This is important also in the light of new treatment opportunities for SLE (Stohl and Hilbert, 2012) and personalized medicine which seems to be just around the corner (Kalunian and Joan, 2009). The ultimate goal is to develop a panel of serological markers that are able to predict SLE flares which then can be prevented by initiating the appropriate treatment. Especially life threatening complications such as kidney failure and transplantation should be prevented. Recently it has been described that anti-chromatin (anti-nucleosome) antibodies are a promising serological marker to help to predict the need for kidney transplantation (Stinton et al., 2007). Whether testing for anti-C1q antibodies in a multiplex setting has the potential to contribute to the improved management of LN patients remains a matter of further research.

## Pathogenic Consequences of Anti-C1q Autoantibodies

Several studies have addressed the mechanisms by which anti-C1q autoantibodies may contribute to tissue damage, especially in LN. Immune complexes eluted from affected glomeruli of human patients and experimental animals revealed that there is a strong enrichment of anti-C1q antibodies and that this deposition seemed to occur via solid-phase C1q (Uwatoko et al., 1991; Mannik and Wener, 1997; Trouw et al., 2004b). Next to the previously mentioned clinical association studies also *in vitro* and *in vivo* animal studies have been performed (Siegert et al., 1992b; Hogarth et al., 1996; Trouw et al., 2004a,b; Bigler et al., 2011). Several of the mouse models of lupus are characterized by a progressive autoimmune disease in which autoantibodies are generated, immune complexes are formed followed by the occurrence of severe glomerulonephritis. Depending on the mouse model these autoimmune phenomena may evolve in different degrees of severity and at different ages. Using MRL/lpr, BXSB, and NZB/W mice, with a severe lupus phenotype, it was demonstrated that anti-C1q autoantibodies are also present in mice and that an increase in the titer of anti-C1q antibodies are associated with the onset of nephritis (Hogarth et al., 1996; Trouw et al., 2004b). Using a different model, using MRL/MpJ^+/+^ mice with a less severe lupus phenotype, it was concluded that glomerulonephritis may also occur in the absence of anti-C1q antibodies (Bigler et al., 2011). In a more experimental setting, injection of rabbit anti-mouse C1q antibodies resulted in immune-complex deposition of C1q and anti-C1q antibodies but the limited degree of deposition was insufficient to induce glomerulonephritis (Trouw et al., 2003). However, injection of mouse anti-mouse C1q autoantibodies into animals that have C1q containing immune complexes in the glomeruli, resulted in strong glomerulonephritis (Trouw et al., 2004a). Collectively these data indicate that anti-C1q antibodies can be present in healthy subjects (mouse or human) which might induce limited deposition in the kidney but no nephritis. Only in the presence of C1q containing immune complexes in the kidney, anti-C1q autoantibodies will amplify the local complement activation and cellular influx resulting in glomerulonephritis. A similar process may also be operational in post-streptococcal glomerulonephritis where anti-C1q autoantibodies were also found to associate with a worse disease course (Kozyro et al., 2008). Why anti-C1q autoantibodies would predominantly enhance the tissue damage in glomeruli and not or less pronounced in other tissues known to contain immune complexes in lupus is currently unknown. The observation that anti-C1q autoantibodies may specifically target C1q bound to early-apoptotic cells (Bigler et al., 2009) raises the question what the *in vivo* consequences would be of enhanced complement activation on apoptotic cells. One possible scenario could be that the natural mechanisms that would limit excessive complement activation on dying cells would be overruled (Trouw et al., 2007, 2008) resulting in lysis of the cells and exposure of autoantigenic components to the immune system. The observation that anti-C1q autoantibodies are also observed in autoimmune thyroid diseases and that their levels correlate with thyroid function (Potlukova et al., 2008) may suggest that the effect of anti-C1q antibodies amplifying immune-complex mediated damage only in the kidney is incomplete and that the presence of anti-C1q antibodies may enhance tissue damage in several other, unexpected clinical conditions.

In conclusion; anti-C1q autoantibodies play an important role in the clinical management of LN. Testing for anti-C1q autoantibodies in large well defined cohorts of several diseases, preferable in a prospective study design, is likely to provide additional clinical conditions for which the testing for anti-C1q autoantibodies would have clinical implications.

## Conflict of Interest Statement

Dr. M. Mahler is employee of INOVA Diagnostics INC., an autoimmune diagnostics company that provides assays for autoantibody detection. He was invited by Dr. L.A. Trouw to participate because of his knowledge of the various commercial assays available for the detection of this autoantibody.
